# Identifying potential inflammatory therapeutic targets and drug candidates in small fiber neuropathy: integrating Mendelian randomization, experimental validation, and deep learning

**DOI:** 10.3389/fnins.2026.1781396

**Published:** 2026-03-24

**Authors:** Guangyu Cai, Jiawei Zheng, Changmao Jiang, Zhu Wei, Shichao Zhang, Xiaohua Zou, Yimin Ren

**Affiliations:** 1 College of Anesthesia, Guizhou Medical University, Guiyang, Guizhou, China; 2 Department of Anesthesiology, Guizhou Provincial People’s Hospital, Guiyang, Guizhou, China; 3 Center for Tissue Engineering and Stem Cell Research of Guizhou Medical University, Guiyang, Guizhou, China; 4 Guizhou University of Traditional Chinese Medicine, Guiyang, Guizhou, China; 5 School of Biology and Engineering, Guizhou Medical University, Guiyang, Guizhou, China; 6 Department of Anesthesiology, The Affiliated Hospital of Guizhou Medical University, Guiyang, Guizhou, China; 7 Guizhou Medical University Key Laboratory of Anesthesia and Pain Mechanism Research, Guiyang, Guizhou, China

**Keywords:** circulating inflammatory proteins, deep learning, Mendelian randomization analysis, molecular docking, neuropathic pain, small fiber neuropathy

## Abstract

**Objective:**

This study aimed to investigate the causal associations between circulating inflammatory proteins and small fiber neuropathy (SFN) by integrating Mendelian randomization (MR) analysis with experimental validation in animal models, and to explore their potential as therapeutic targets.

**Methods:**

A two-sample bidirectional MR analysis was conducted to evaluate the genetic causal associations between 91 inflammatory proteins and SFN. A paclitaxel-induced SFN mouse model was developed to assess behavioral changes, intraepidermal nerve fiber density, and the expression levels of key inflammatory factors in serum, dorsal root ganglia, and spinal cord. Computational drug screening using deep learning (TransformerCPI 2.0) combined with molecular docking analysis screened small-molecule candidates with high predicted interaction likelihood to target proteins.

**Results:**

MR analysis nominated suggestive associations of C-C motif chemokine ligand 11 (CCL11, odds ratio (OR) = 1.460, 95% confidence interval (CI) = 1.059–2.012, *p* = 0.021) and interleukin 18 receptor 1 (IL18R1, OR = 1.186, 95% CI = 1.011–1.391, *p* = 0.036) with increased SFN risk, whereas monocyte chemotactic protein 2 (MCP2) showed a suggestive protective association (OR = 0.842, 95% CI = 0.731–0.970, *p* = 0.017). However, after Benjamini–Hochberg false discovery rate correction across 91 proteins, none of these associations remained significant. In a murine model, paclitaxel administration induced mechanical hypersensitivity and resulted in a reduction of intraepidermal nerve fiber density. Elevated expression levels of CCL11, MCP2, IL18R1 in affected tissues were observed. Utilizing deep learning and molecular docking techniques, several small-molecule compounds with high binding affinity to these inflammatory targets were screened, indicating their potential as candidate compounds for future therapeutic development.

**Conclusion:**

CCL11 and IL18R1 are suggested as potential inflammatory targets in SFN. MCP2 showed discordant genetic and experimental signals, which may reflect context-dependent regulation and differences between genetically predicted long-term effects and acute injury responses. This study applies an integrative framework that integrates genetic prediction, experimental validation, and drug discovery, providing novel insights into SFN pathogenesis and generates hypotheses for future intervention.

## 1 Introduction

Small fiber neuropathy (SFN) arises from damage to thinly myelinated Aδ-fibers and unmyelinated C fibers. This pathological alteration may be the outcome of metabolic disturbances, drug toxicity, immune mediation, or genetic factors. It may affect small sensory fibers, autonomic nerve fibers, or both, leading to non-length-dependent pain and paresthesias, autonomic neuropathy, or a combination of these symptoms (Gross and Üçeyler, 2020; Oaklander and Nolano, 2019; Terkelsen et al., 2017; Devigili et al., 2020; Tavee, 2022). The symptoms of SFN are varied and intricate, including burning, stabbing pain, cramps, electric shocks, and itching. Patients may also suffer from orthostatic hypotension, dry mouth, gastrointestinal or sexual dysfunction, dry eyes, altered sweating, and urinary complications, all of which can greatly impact their quality of life (Timmins et al., 2020; Terkelsen et al., 2017; Devigili et al., 2020). Research indicates that early detection and intervention for SFN can enhance disease management strategies and improve outcomes for diabetic patients (Sharma et al., 2022). Studies have demonstrated that small nerve fibers possess a high regenerative capacity, and timely diagnosis coupled with early treatment can result in significant regeneration and recovery from neuropathy (Woolf and Salter, 2000; Bautista et al., 2020). Nevertheless, the incomplete understanding of SFN pathogenesis and its inherent complexity render both diagnosis and treatment challenging (Terkelsen et al., 2017; Timmins et al., 2020; Raasing et al., 2021; Kelley and Hackshaw, 2021; Devigili et al., 2020).

While the exact pathogenic mechanisms of SFN remain unclear, research indicates that inflammatory processes contribute to its development. Pro-inflammatory and anti-inflammatory proteins modulate skin nociceptors and significantly contribute to SFN-related pain (Kreß et al., 2021). Studies analyzing protein expression in leukocytes of SFN patients reveal elevated systemic levels of interleukin-8, interleukin-2, and tumor necrosis factor compared to healthy controls (Kreß et al., 2023). The role of inflammation is vital in the progression of chemotherapy-induced peripheral neuropathy (Jia et al., 2025; Gupta et al., 2022). Inflammation might play a crucial role in SFN development; however, the causal relationships between circulating inflammatory proteins and SFN remain uncertain.

Mendelian randomization (MR) uses single-nucleotide polymorphisms (SNPs) as instrumental variables to examine causal relationships between exposures and outcomes (Burgess et al., 2019). This analytical method harnesses the random allocation of SNPs, employing them as instrumental variables in the analysis. During meiosis, alleles are randomly distributed, and non-allelic genes segregate independently. As a result, MR effectively addresses confounding variables and reverse causality, enhancing research reliability and establishing itself as a robust causal inference method. This study employed summary data from genome-wide association studies (GWAS) on circulating inflammatory proteins and SFN to explore their causal relationship using a two-sample MR analysis. To ensure the robustness of our findings, we performed sensitivity analyses, reverse causality testing, Bayesian colocalization, and a phenome-wide association study (PheWAS). We constructed a protein–protein interaction (PPI) network to investigate the functional connections among the identified targets (Szklarczyk et al., 2023), and explored potential therapeutic strategies using deep learning-based drug prediction and molecular docking (Trott and Olson, 2010; Chen et al., 2023).

Chemotherapy-induced peripheral neuropathy (CIPN) is a frequent dose-limiting adverse effect of cancer treatment that significantly compromises patients’ quality of life and several chemotherapeutic agents are established triggers of SFN (Timmins et al., 2020). We developed a mouse model of paclitaxel-induced SFN and investigated its characteristic pathological features through behavioral assessments and measurements of intraepidermal nerve fiber density. Based on the targets prioritized by the Mendelian randomization analysis, we then used enzyme-linked immunosorbent assays (ELISAs) to quantify their levels in serum, dorsal root ganglia (DRG), and spinal cord tissues, providing experimental support for the MR-based inflammatory target prioritization.

Recent advancements in deep learning have significantly enhanced the prediction of compound-protein interactions. The TransformerCPI framework, leverages transformer self-attention mechanisms to facilitate end-to-end predictions using solely sequence information, demonstrating superior performance compared to traditional methods across multiple benchmark datasets (Chen et al., 2023). This approach obviates the necessity for three-dimensional structural data and reduces false-positive predictions through label reversal training, thereby offering a robust computational platform for virtual screening. In this study, we used the model to screen small molecules from the TargetMol database against Mendelian randomization–prioritized inflammatory targets, and then performed molecular docking to explore plausible binding poses and to support subsequent candidate selection.

By integrating Mendelian randomization, experimental validation, and deep learning–based screening, this study investigates inflammatory mechanisms in SFN, provides mechanistic insights, and identifies suggestive candidate compounds for future validation and therapeutic exploration.

## 2 Materials and methods

### 2.1 Study design

The genetic analysis phase comprised two-sample MR using GWAS summary statistics for 91 circulating inflammatory proteins as exposures and SFN as the outcome, with genome-wide SNPs selected as instrumental variables, to investigate causal relationships between inflammatory proteins and SFN. Complementary analyses encompassed sensitivity analysis, Bayesian colocalization to verify shared causal variants, phenome-wide MR to assess trait specificity, and protein–protein interaction network construction. For experimental corroboration, a paclitaxel-induced SFN mouse model was developed, evaluating mechanical hypersensitivity via von Frey testing, intraepidermal nerve fiber density through skin biopsies, and target protein expression in serum, dorsal root ganglia, and spinal cord using ELISA and immunohistochemistry. The drug discovery phase utilized deep learning-based virtual screening (TransformerCPI 2.0) to predict compound-protein interactions, followed by molecular docking and affinity scoring to identify high-priority small-molecule compounds.

### 2.2 MR assumptions

**Figure 1:**
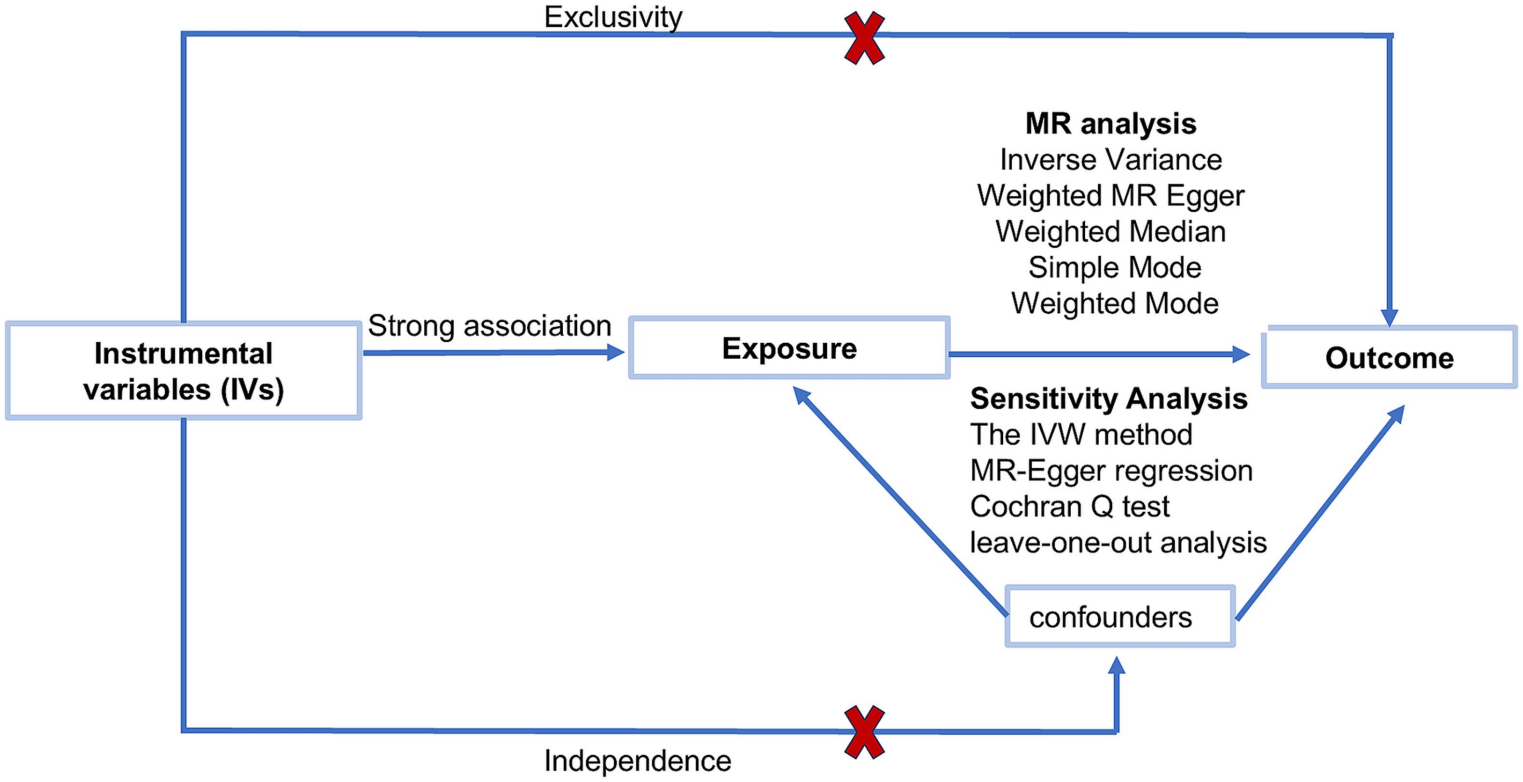
Study workflow and Mendelian randomization (MR) analysis pipeline. The diagram summarizes instrument selection, primary MR analyses, and sensitivity analyses.

To examine the potential causal connection between 91 circulating inflammatory proteins and SFN, bidirectional MR analyses were carried out (Figure 1). This study follows the three core assumptions of traditional MR analysis: association, independence, and exclusivity. The association assumption requires that the instrumental variables in the study must have a strong correlation with the exposure of interest. The independence assumption suggests that there is no link between single-nucleotide polymorphisms (SNPs) and the outcome through confounding pathways. The exclusivity assumption asserts that the impact of instrumental variables on the outcome is mediated only by the exposure, with no direct effects (Emdin et al., 2017).

### 2.3 Sources of data

We employed GWAS data from 14,824 European participants to analyze genetic variations linked to 91 circulating inflammatory proteins (Zhao et al., 2023). Genetic variants associated with small fiber neuropathy were sourced from the FINNGEN database R11 version^1^ (Kurki et al., 2023), which comprised 786 cases and 445,865 controls. Data for this study were obtained from datasets that are both publicly accessible and previously published.

### 2.4 Instrumental variable selection method

To investigate the causal relationship between inflammatory proteins and SFN, we selected instrumental variables based on specific criteria: for inflammatory proteins as the exposure, SNPs with a strong association (*p* < 5 × 10^−6^) were chosen, while for SFN as the exposure, a threshold of *p* < 1 × 10^−5^ was used to ensure an adequate SNP count. SNPs in linkage disequilibrium, defined by a distance limit of 10,000 kb and an *r*^2^ value less than 0.001, were excluded. SNPs that are palindromic were not included; additionally, SNPs with an *F*-statistic lower than 10, indicating weak instruments, were taken out of the analysis. The number of instrumental SNPs for each exposure, together with full instrument details and F-statistics, are provided in Supplementary Table S1. Significantly associated SNPs with the outcome or confounding factors were manually excluded using the LDtrait Tool.^2^

### 2.5 Sensitivity analysis

Heterogeneity was assessed using Cochrane’s Q test, with significance set at a *p*-value below 0.05. To examine potential pleiotropic effects of SNPs acting as instrumental variables, MR-Egger regression was applied, with a *p*-value above 0.05 suggesting no horizontal pleiotropy (Burgess and Thompson, 2017). The MR-PRESSO test was used to detect and measure pleiotropic effects, identify outliers impacting study results, and assess improvements after their removal. A Leave-one-out sensitivity analysis was conducted by systematically removing individual SNPs to evaluate their influence on circulating inflammatory proteins or SFN and to assess the robustness of the findings.

### 2.6 Bayesian colocalization analysis

We performed colocalization analysis using the R package “coloc” to identify possible common genetic variants with causal effects located physically between the detected inflammatory proteins and SFN. Bayesian colocalization analysis evaluates posterior probabilities for five hypotheses: PPH0 indicates no association with either inflammatory proteins or SFN; Posterior Probability of Hypothesis1 (PPH1) suggests association with inflammatory proteins only; PPH2 implies association with SFN only; PPH3 denotes association with both inflammatory proteins and SFN but with separate causal variants; PPH4 signifies association with both inflammatory proteins and SFN, sharing a common causal variant. Evidence of strong colocalization is defined as PPH4 ≥ 0.75, while moderate colocalization is indicated by 0.5 < PPH4 < 0.75.

### 2.7 Analysis of phenome-wide associations

To further assess potential horizontal pleiotropy of the prioritized targets and to screen for possible on-target safety liabilities, we conducted a phenome-wide association study (PheWAS) using the AstraZeneca PheWAS Portal.^3^ This resource provides association results for approximately 15.5 K binary and 1.5 K continuous phenotypes derived from a subset of ~450,000 United Kingdom (UK) Biobank participants with exome sequencing data, as described in the original publication (Wang et al., 2021). We queried the candidate targets on the portal and interpreted phenome-wide signals using the portal’s default multiple-testing threshold (*p* < 2 × 10^−9^) to control for false positives.

### 2.8 Protein–protein interaction network construction

We examined the interactions between screened inflammatory proteins and potential mechanisms underlying SFN by retrieving the top 500 SFN-related therapeutic targets from the GeneCards database. Then we selected SFN-related therapeutic targets interacting with the target inflammatory proteins from them. We performed a PPI analysis to explore the functional relationships and potential biological pathways between inflammatory proteins and therapeutic targets. We conducted PPI analysis via the STRING database and Cytoscape software (Szklarczyk et al., 2023; Doncheva et al., 2023), setting a minimum interaction score of 0.4 for significant interactions.

### 2.9 Experimental reagents

Paclitaxel (B21695, Shanghai, China) was purchased from Yuanye Biotechnology Co., Ltd. The experimental solution was formulated by dissolving paclitaxel in dimethyl sulfoxide (DMSO, GENTIHOLD, Beijing, China), emulsifying it with Tween 80 (Solarbio, Beijing, China), and subsequently diluting it with normal saline in a volumetric ratio of 1:1:8.

### 2.10 Animal models and treatment

This study was approved by the Animal Care and Welfare Committee of Guizhou Medical University and was conducted in compliance with the institution’s guidelines for the care and use of laboratory animals. Furthermore, all animal experiments adhered to the ARRIVE guidelines. C57BL/6J mice were purchased from the Laboratory Animal Center of Guizhou Medical University. Mice were kept in a temperature-controlled facility (23–24 °C, 50–60% humidity) with a 12-h light/dark cycle and unlimited access to food and water. They acclimated for at least 7 days before experiments. Our sample size was based on prior paclitaxel-CIPN behavioral studies, and we therefore used 8 mice per group for the behavioral and histological endpoints in this study (Sankaranarayanan et al., 2023). Sixteen male C57BL/6J (8 weeks old, 20-24 g) mice were randomly assigned to either a control (CTRL) group or a paclitaxel (PTX) group (*n* = 8 mice per group), with the experimental unit being a single mouse. The PTX group was administered paclitaxel at a dosage of 4 mg/kg intraperitoneally every other day, whereas the CTRL group received an equivalent volume of solvent (comprising 10% DMSO, 10% Tween 80, and 80% saline) following the same schedule. A total of four injections were administered.

### 2.11 Behavioral testing

All behavioral assessments were conducted by a single experimenter in a blinded manner. The mechanical paw withdrawal threshold of the left hind paw was assessed using von Frey filaments (0.008 g-1.4 g) by the up-down method. Mice were placed in plastic cages with a metal mesh floor and acclimatized to the testing environment for 1 h prior to testing. The filament was applied perpendicularly to the plantar surface of the hind paw for 3–4 s until a paw withdrawal response was observed. Each trial was separated by a 5-min interval, and the 50% paw withdrawal threshold was calculated. Baseline measurements were recorded 1 day before the initial injection. Additional tests were conducted on days 1, 3, 5, and 7 after the first injection.

### 2.12 ELISA and immunohistochemistry

In our PTX regimen (4 mg/kg i.p. every other day for a total of four injections), tissues were collected on day 8 after the first injection, which is approximately 2 days after the fourth (final) injection. At this time, mechanical hypersensitivity was already robust and persistent (days 3, 5, and 7 after the first injection), indicating that our sampling corresponds to a late initiation/transition-to-chronicity stage with early established (maintenance-like) pain, rather than the initiation-only or resolution phases.

On the eighth day, eight mice from each experimental group were subjected to deep anesthesia using isoflurane. Blood samples were collected via cardiac puncture, followed by transcardial perfusion with phosphate-buffered saline. The L4–L6 spinal cord segments, DRG, and hind paw footpads were promptly harvested for subsequent analysis. Blood samples were maintained at room temperature for 1 h prior to centrifugation to separate serum, which was subsequently stored at −80 °C. Spinal cord and DRG tissues were rapidly frozen in liquid nitrogen. Total protein was extracted from these tissues and quantified utilizing a Bicinchoninic Acid Assay in accordance with the manufacturer’s protocol. Concentrations of C-C motif chemokine ligand 11 (CCL11), interleukin 18 receptor 1 (IL18R1), and monocyte chemotactic protein 2(MCP2) in the serum, spinal cord, and DRG were determined using commercial ELISA kits (KEQIAOBIO, Shanghai, China).

Footpad samples were fixed in 4% paraformaldehyde at 4 °C overnight and then dehydrated in 30% sucrose at 4 °C until they sank. The tissues were sectioned at a thickness of 20 μm using a cryostat. The sections were incubated overnight with a primary antibody against Protein Gene Product 9.5 (PGP9.5, rabbit anti-PGP9.5, 1:100, Abclonal, Wuhan, China), followed by incubation with a secondary antibody (goat anti-rabbit IgG, 1:500, Abcam, Cambridge, UK) for 1 h. Nuclei were stained with 4′,6-Diamidino-2-Phenylindole (DAPI, 1:5000, Solarbio, Beijing, China) for 5 min, and slides were mounted with Mounting Medium (Solarbio, Beijing, China).

### 2.13 Morphological analysis

The density of epidermal nerve fibers was evaluated through skin biopsy, which is an established standard for diagnosing small fiber neuropathy. Images of the skin sections were acquired at 400 × magnification utilizing an Olympus SpinSR10 super-resolution spinning disk confocal microscope (Olympus Corporation, SpinSR10, Tokyo, Japan). Subsequent image processing and analysis were conducted using Cellsens (Olympus) and ImageJ software (version 1.53c, National Institutes of Health). Nerve fibers traversing the dermal–epidermal junction were enumerated, and intraepidermal nerve fiber density (IENFD) was quantified as the number of fibers per millimeter of epidermal length.

### 2.14 Virtual screening of candidate compounds

Candidate small molecules were retrieved from the TargetMol database,^4^ and their chemical structures were obtained for subsequent analyses. Structural data for the proteins CCL11 (UniProt ID: P51671), IL18R1 (UniProt ID: Q13478), and MCP2 (UniProt ID: P80075) were retrieved from the UniProt database.^5^ Virtual screening was conducted utilizing the TransformerCPI 2.0 tool (available at https://github.com/lifanchen-simm/transformerCPI2.0/). The pre-trained “Virtual Screening” model was employed to predict the binding affinities between the small molecule drugs and the target proteins. The TransformerCPI model code was downloaded and installed in the local computing environment following the installation instructions provided on the official website. The preprocessed data of the target protein structures and the Simplified Molecular Input Line Entry System (SMILES) representations of small molecule drugs were systematically organized and formatted to meet the input requirements of the TransformerCPI model. TransformerCPI2.0 was then used to compute model-predicted target–compound interaction scores for each candidate compound–protein pair. The output score ranges from 0 to 1, with higher values indicating a higher predicted likelihood of interaction/binding. In this study, the model outputs were used only for computer-aided prioritization of candidate compounds and should not be considered direct evidence of experimental binding affinity or therapeutic efficacy.

### 2.15 Molecular docking

AutoDock Vina 1.2.2, a computational tool for protein-ligand docking, was utilized to assess the binding energy and interaction patterns between the candidate drugs and their respective targets. The top three drugs screened through virtual screening by the TransformerCPI model were selected for molecular docking with three inflammatory proteins. The molecular structures of the selected drugs were retrieved from the PubChem compound database,^6^ while the structures of CCL11 (Protein Data Bank (PDB) ID: 7SCS), MCP2 (PDB ID: 1ESR), and IL18R1 (PDB ID: 3WO4) were obtained from the Protein Data Bank.^7^ Molecular docking studies were conducted utilizing AutoDock Vina version 1.2.2 (available at http://autodock.scripps.edu/), followed by model visualization. A binding energy below −5.0 kcal/mol is indicative of favorable binding activity between the ligand and receptor, whereas a binding energy below −7.0 kcal/mol signifies strong binding affinity.

### 2.16 Statistical analysis

All Mendelian randomization analyses were performed using R software version 4.4.1.^8^ We utilized the R packages TwoSampleMR (v0.6.8), MR-PRESSO (v1.0), MendelianRandomization (v0.10.0), and ggplot2 (v3.5.1), along with their associated functions. Inverse Variance Weighted (IVW) method was chosen as the main analytical technique due to its robustness in estimating causal effects from observational data. Statistical significance was determined at a *p*-value threshold of less than 0.05. To account for multiple testing across 91 proteins, *p* values from the primary MR analysis were adjusted using the Benjamini–Hochberg false discovery rate (BH-FDR) procedure. Associations with *q* < 0.05 were considered statistically significant. Our analysis also employed various methods such as Simple Mode, Weighted Median, MR Egger regression and Weighted Mode. The causal relationship between the 91 circulating inflammatory proteins and SFN was expressed using the odds ratio (OR) and its 95% confidence interval (CI).

Statistical analyses of experimental validation were executed using GraphPad Prism version 10.4.1, with data presented as mean ± standard error of the mean (SEM). Behavioral data, specifically the mechanical withdrawal threshold, were analyzed using a two-way repeated measures analysis of variance (RM-ANOVA), with group serving as the between-subjects factor and time as the within-subjects factor. Normality was assessed using the Shapiro–Wilk test. When sphericity could not be assumed, the Geisser–Greenhouse correction was applied. Effect sizes for RM-ANOVA main effects and interaction were quantified using partial eta squared (ηp^2^), and exact F statistics with corresponding degrees of freedom were reported. In instances of significant interaction, Sidak’s *post hoc* test was employed. Comparisons of inflammatory protein levels and intraepidermal nerve fiber density between the two groups were performed by t-test. Statistical significance was determined at a p-value threshold of less than 0.05.

## 3 Results

### 3.1 Causal effect of inflammatory proteins on SFN

**Figure 2:**
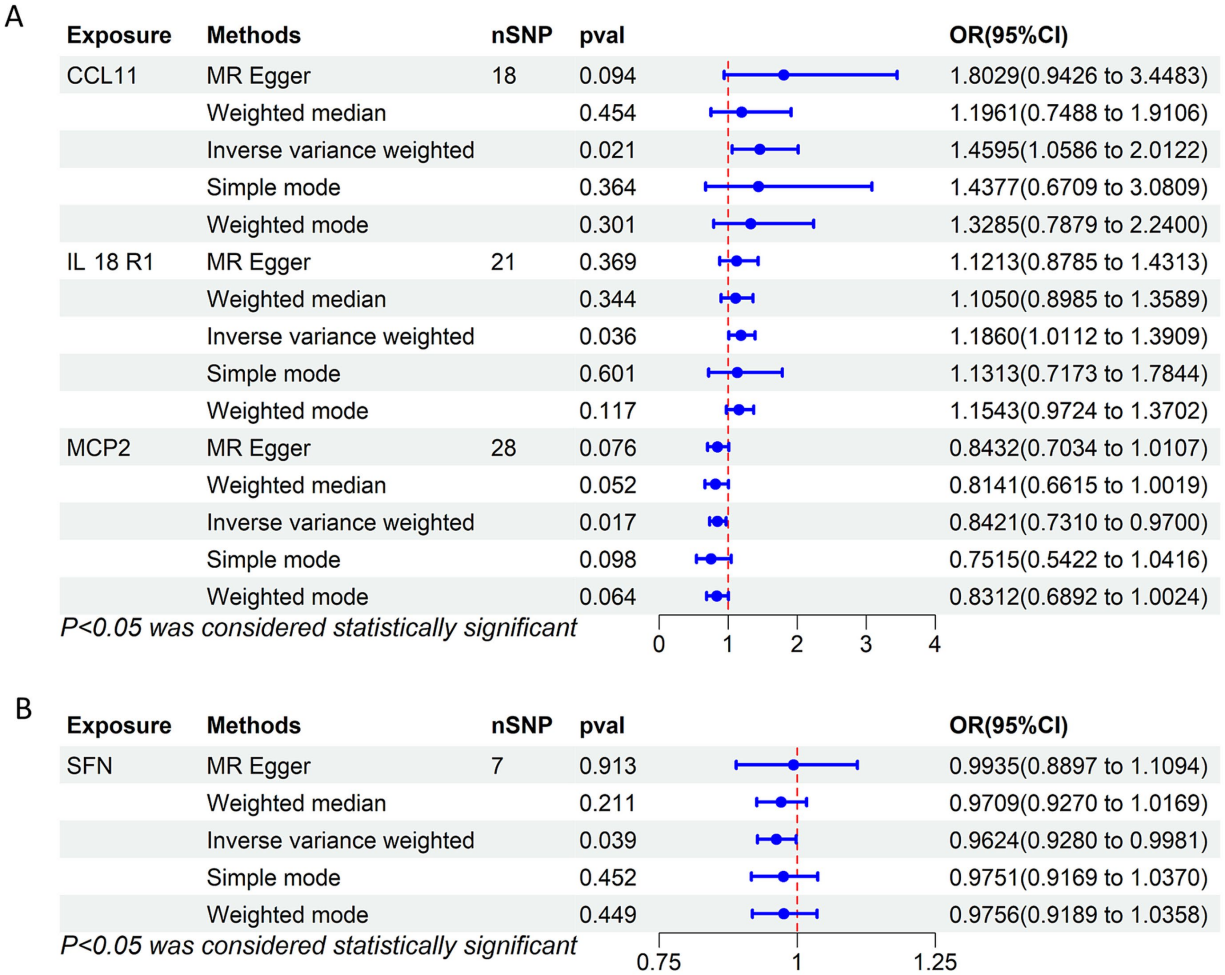
Forest plots of bidirectional MR results. **(A)** MR estimates for the effects of circulating inflammatory proteins on small fiber neuropathy (SFN). **(B)** Reverse MR estimates for the effect of SFN on circulating inflammatory proteins. CCL11, C-C motif chemokine ligand 11; IL18R1, interleukin-18 receptor 1; MCP2, monocyte chemoattractant protein 2.

**Figure 3:**
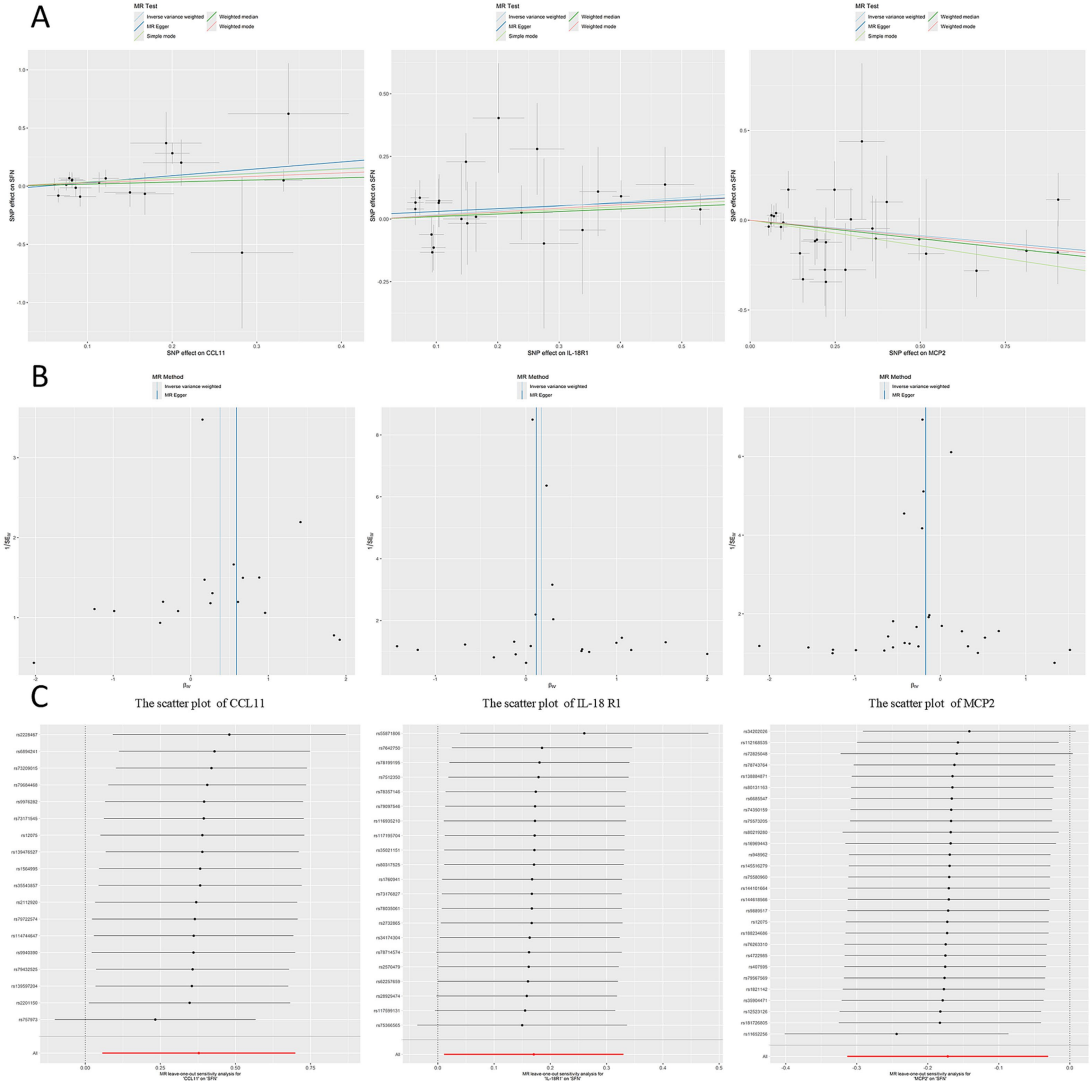
MR analyses for three prioritized inflammatory proteins and SFN. **(A)** Scatter plots showing SNP-specific associations with exposure (protein) and outcome (SFN) with MR regression lines. **(B)** Funnel plots assessing potential directional pleiotropy/heterogeneity. **(C)** Leave-one-out analyses evaluating the influence of individual SNP instruments on the MR estimate.

The MR analysis suggested MCP2 as a protective factor and CCL11 and IL18R1 as risk factors when evaluating 91 circulating inflammatory proteins as exposures. Specifically, higher genetically predicted MCP2 levels were associated with a lower risk of SFN (*p* = 0.017, OR = 0.842, 95%CI = 0.731–0.970), whereas higher CCL11 (*p* = 0.021, OR = 1.460, 95%CI = 1.0589–2.012) and IL18R1 (*p* = 0.036, OR = 1.186, 95%CI = 1.011–1.391) levels were associated with an increased risk of SFN (Figure 2A). After BH-FDR correction across 91 proteins, none of these associations remained significant (MCP2 *q* = 0.883; CCL11 *q* = 0.883; IL18R1 *q* = 0.883), and therefore they should be interpreted as suggestive signals. Each SNP’s F-statistic surpassed 10, showing a small probability of instruments with low strength bias (Supplementary Table S1). Figure 3A presents scatter plots of the MR analysis. Supplementary Table S1 provides detailed characteristics of the SNPs.

### 3.2 Causal effect of SFN on inflammatory proteins

**Figure 4:**
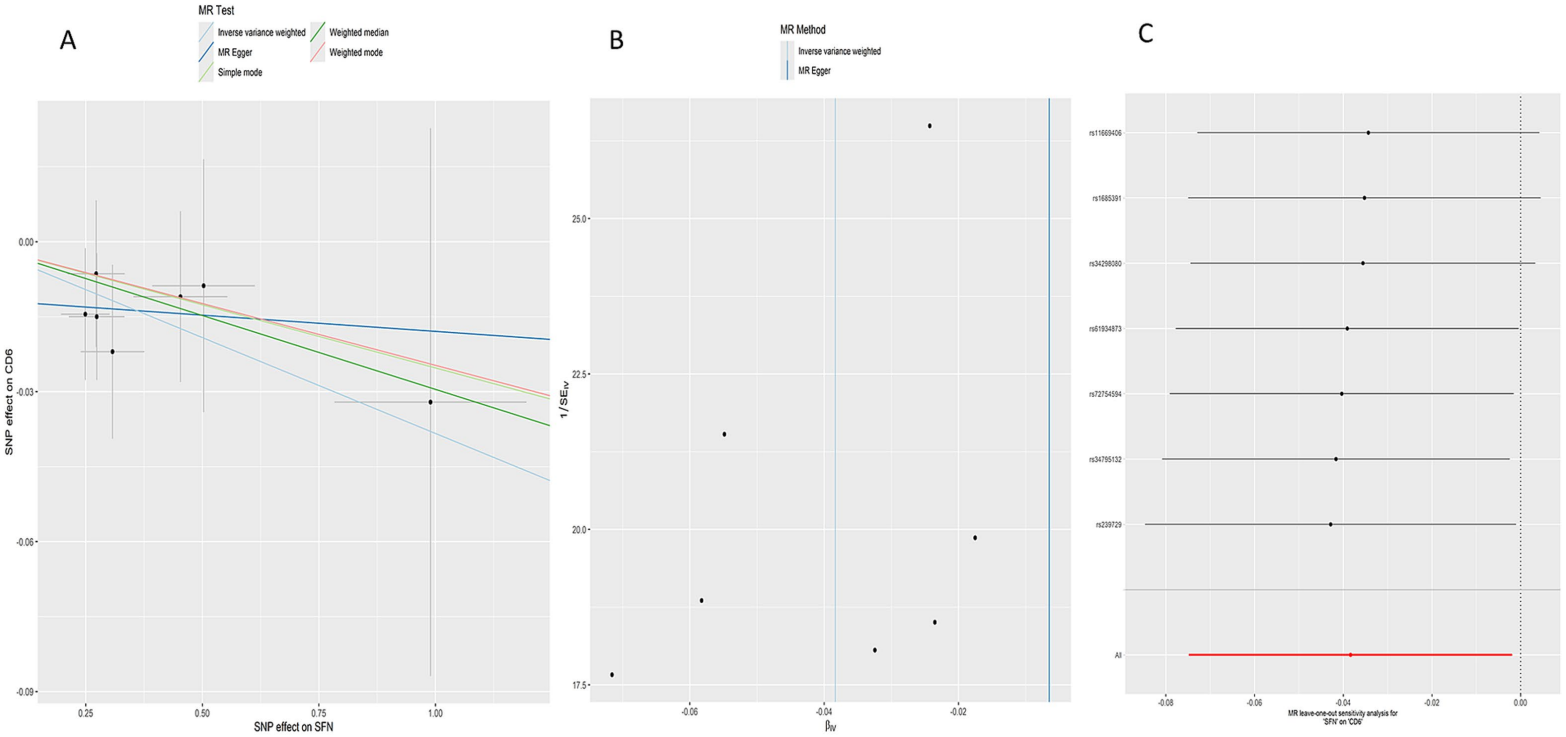
Reverse MR analysis of SFN on CD6 (T-cell differentiation antigen CD6). **(A)** Scatter plot **(B)** Funnel plot **(C)** Leave-one-out analysis.

In the reverse analysis, MR analysis indicated that SFN decreased T-cell differentiation antigen CD6 levels (*p* = 0.039, OR = 0.963, 95% CI = 0.928–0.998) without significantly affecting other circulating inflammatory proteins (Figure 2B). Figure 4A presents scatter plots illustrating the MR analysis. Supplementary Table S1 provides detailed information on the SNPs.

### 3.3 Sensitivity analysis

As depicted in the figures, in our bidirectional Mendelian analysis, the funnel plot demonstrated a symmetrical funnel shape (Figures 3B, 4B). Cochrane’s *Q* test revealed no heterogeneity among SNPs in the bidirectional Mendelian randomization analysis (*p* > 0.05; Table 1). The MR-Egger analysis indicated an absence of horizontal pleiotropy (*p* > 0.05), which was supported by the MR-PRESSO test, affirming the reliability of our findings (Table 1). We conducted a leave-one-out analysis, revealing that no individual SNP significantly influenced the results (Figures 3C, 4C).

**Table 1:** Tests for heterogeneity and horizontal pleiotropy in the bidirectional Mendelian randomization analysis.

Exposure	Heterogeneity tests		Pleiotropy test				
	The Cochrane’s *Q* tests		MR-Egger regression			MR-PRESSO global test	
	*Q*	*p*-value	Egger_ intercept	se	*p*-value	*p*-value	Outlier-corrected
The causal effect of inflammatory proteins on SFN							
CCL11	17.881	0.396	−0.028	0.038	0.471	0.405	NA
IL18 R1	18.674	0.543	0.019	0.031	0.559	0.591	NA
MCP2	26.009	0.518	−0.0005	0.023	0.982	0.521	NA
The causal effect of SFN on CD6							
SFN	1.008	0.985	−0.011	0.019	0.575	0.989	NA

SFN, small fiber neuropathy; CCL11, C-C motif chemokine ligand 11; IL18 R1, Interleukin 18 receptor 1; MCP2, Monocyte chemotactic protein 2; CD6, T-cell differentiation antigen CD6.

### 3.4 Bayesian colocalization analysis and phenome-wide association study

Colocalization analysis revealed a PPH4 value greater than 0.5 for CCL11, indicating moderate colocalization results (Table 2; Supplementary Figure S1).

**Table 2:** Bayesian co-localization analysis for three possible causal proteins.

Protein	SNP	PPH0	PPH1	PPH2	PPH3	PPH4
CCL11	rs757973	0.000	0.291	0.000	0.097	0.612
MCP2	rs2548023	0.563	0.152	0.026	0.007	0.251
IL18R1	rs28929474	0.001	0.847	0.000	0.051	0.100

Evidence of strong colocalization is defined as Posterior Probability of Hypothesis4 (PPH14) ≥ 0.75, while moderate colocalization is indicated by 0.5 < PPH4 < 0.75. SNP, single-nucleotide polymorphism.

Using the AstraZeneca PheWAS Portal, which includes 17,361 binary and 1,419 quantitative phenotypes, we performed a genetic-level PheWAS to examine whether the prioritized proteins show broader associations that might reflect horizontal pleiotropy or potential safety liabilities. The PheWAS results provide an overview of the relationships between genetically predicted protein levels and a wide range of traits. As shown in Supplementary Figure S2, we did not observe significant genetic associations between the three target proteins and other disease traits under the portal’s phenome-wide significance threshold. Detailed results are provided in Supplementary Tables S2–S4.

### 3.5 Protein–protein interaction network construction

**Figure 5:**
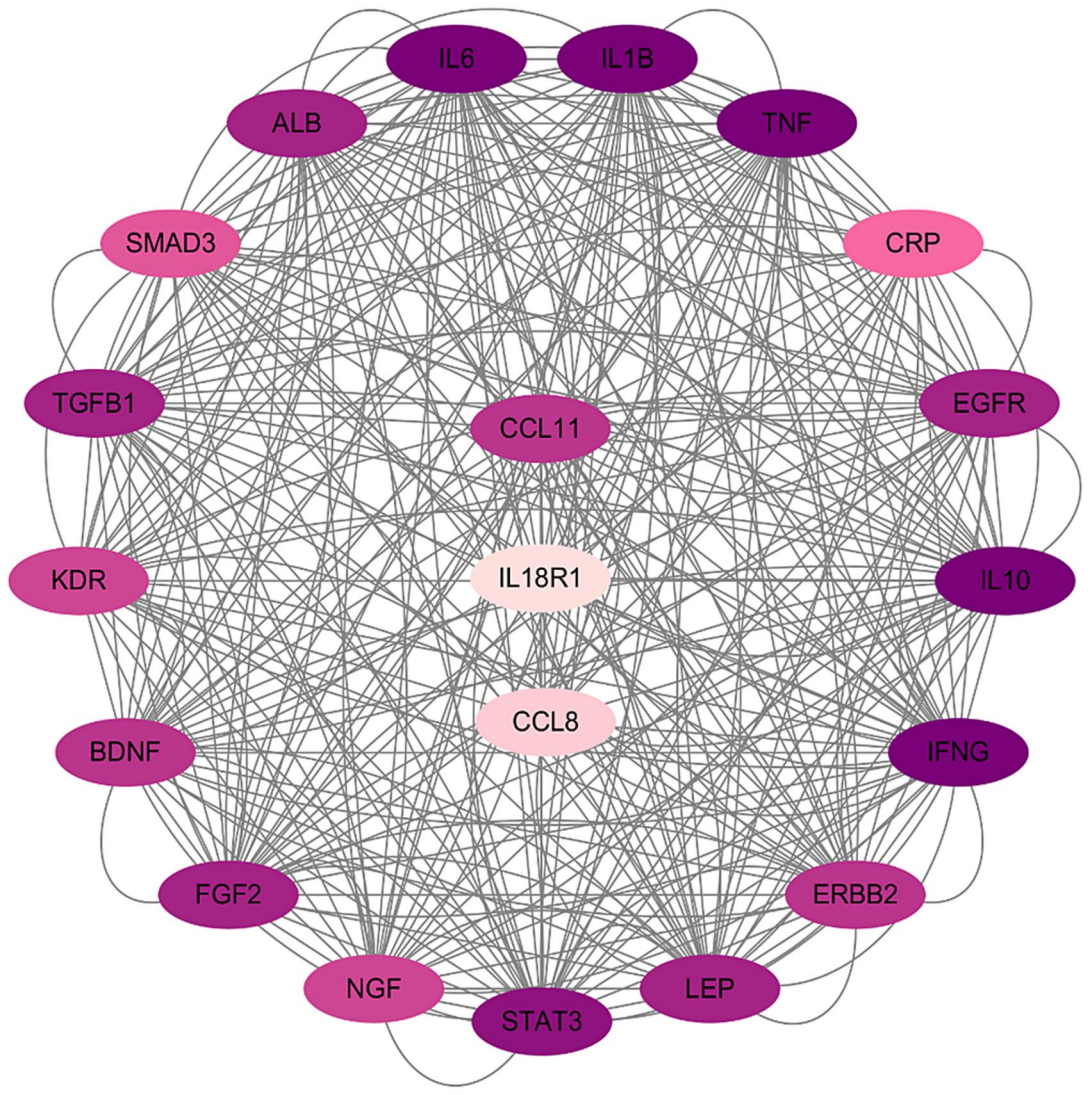
Protein–protein interaction (PPI) network of inflammatory proteins and SFN-related targets. Nodes represent proteins and edges represent known or predicted interactions; darker node shading indicates stronger connectivity/interaction strength. CCL8, C-C motif chemokine ligand 8 (also known as monocyte chemoattractant protein 2, MCP2).

The STRING database was used to load SFN-related therapeutic targets along with the three protein targets to develop a PPI network. The outcomes are illustrated in the figure (Figure 5), and the PPI network reveals interactions among them. CCL11 demonstrates significant interactions with therapeutic targets associated with SFN.

### 3.6 Paclitaxel-induced mechanical allodynia, small fiber neuropathy, and inflammatory factor changes

Based on the MR results, we screened CCL11, IL18R1, and MCP2 as prioritized candidate targets for downstream analyses. We then evaluated these targets in a paclitaxel-induced mouse model and further conducted computer-aided compound screening (deep learning–based scoring and molecular docking) to support candidate compound selection.

**Figure 6:**
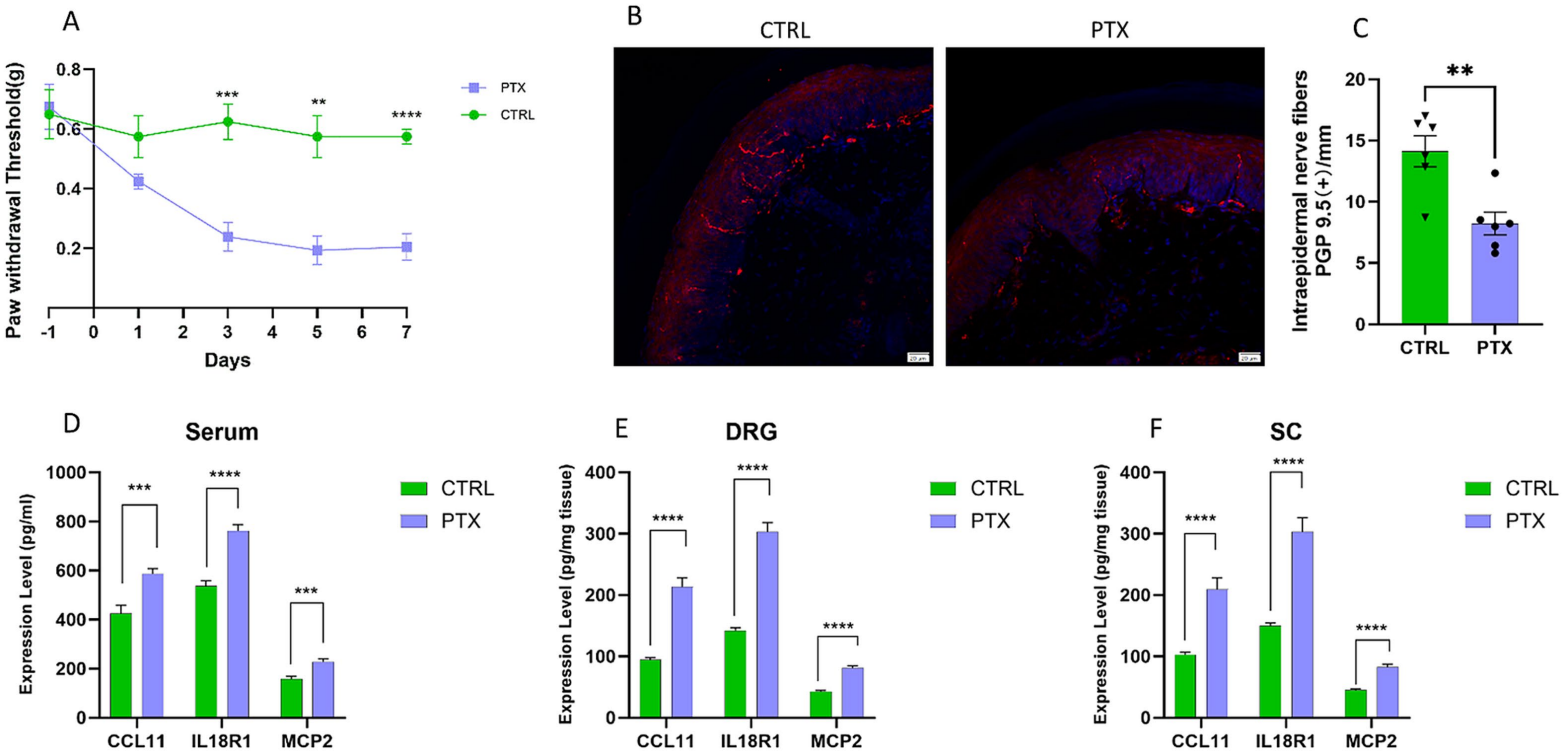
Paclitaxel-induced small fiber neuropathy (SFN) and associated inflammatory changes in mice. **(A)** Mice receiving paclitaxel (PTX) developed persistent mechanical hypersensitivity on days 3, 5, and 7 after the first injection compared with controls (*n* = 8 per group). Data distribution was visually inspected and no marked deviation from normality was observed. Two-way repeated-measures ANOVA showed significant main effects of group (*F*(1,14) = 16.40, *p* = 0.00119, ηp^2^ = 0.540) and day (Geisser–Greenhouse corrected: *ε* = 0.7474; *F*(2.990,41.85) = 14.95, *p* = 9.49 × 10^−7^, ηp^2^ = 0.516), as well as a significant Group × Day interaction (*F*(4,56) = 9.564, *p* < 0.0001, ηp^2^ = 0.406; GG-corrected *p* = 6.33 × 10^−5^). Sidak’s multiple comparisons indicated significant differences between PTX and CTRL from day 3 onward (adjusted *p* = 0.0010, 0.0034, and <0.0001 for days 3, 5, and 7, respectively). **(B)** Representative hind paw skin sections stained for PGP9.5 (red) to visualize intraepidermal nerve fibers and DAPI (blue) to label nuclei and delineate the dermal–epidermal junction. **(C)** Quantification of intraepidermal nerve fiber density (IENFD; fibers/mm). Nerve fibers crossing the dermal–epidermal junction were counted and normalized to epidermal length. Three randomly selected sections per mouse were analyzed and averaged (*n* = 6 per group). The PTX group showed reduced IENFD compared with CTRL. **(D–F)** Levels of CCL11, IL18R1, and MCP2 in serum **(D)**, dorsal root ganglia (DRG) **(E)**, and spinal cord **(F)** in CTRL and PTX mice (*n* = 8 per group). Data are presented as mean ± SEM. CTRL, control; PTX, paclitaxel; DRG, dorsal root ganglia; SC, spinal cord. **p < 0.01, ***p < 0.001, ****p < 0.0001.

To assess the impact of paclitaxel on peripheral nerve function, we initially examined alterations in the mechanical pain threshold in mice through behavioral assays. In comparison to the control group, the PTX-treated group demonstrated persistent mechanical allodynia on days 3, 5, and 7 following the initial administration (Figure 6A), indicating that paclitaxel induced pain behaviors associated with peripheral neuropathy. Morphological alterations in intraepidermal nerve fibers within the hind paw skin of mice were evaluated via immunofluorescence staining. PGP 9.5 staining (red) revealed the presence of nerve fibers at the dermal-epidermal junction (Figure 6B). Quantitative analysis indicated a significant reduction in IENFD in the PTX group compared to the CTRL group (Figure 6C), suggesting that paclitaxel induced small fiber neuropathy in the mouse hind paw skin. To investigate the potential immune-inflammatory mechanisms underlying paclitaxel-induced small fiber neuropathy, we quantified the expression levels of the inflammatory factors CCL11, IL18R1, and MCP2 in serum, DRG, and spinal cord using ELISA. The findings indicated that the serum concentrations of CCL11, IL18R1, and MCP2 in the PTX group were significantly elevated compared to those in the CTRL group (Figure 6D). A similar upregulation of these three inflammatory markers was also observed in the DRG (Figure 6E) and spinal cord (Figure 6F) of the PTX group.

### 3.7 Drug virtual screening and molecular docking results

We applied TransformerCPI2.0 to generate predicted interaction scores between candidate compounds and the prioritized inflammatory targets (CCL11, IL18R1, and MCP2), and used these scores to rank compounds for follow-up in silico analyses. The top three scoring compounds for each target were selected for further evaluation. As shown in Table 3, the selected compounds yielded scores >0.97, indicating high predicted interaction likelihood within this model. Subsequent molecular docking analyses revealed that most candidate drugs demonstrated favorable binding activity with their respective targets. Among the compounds analyzed, Dracorhodin perchlorate and Pseudolaric acid A-O-b-D-glucopyranoside exhibited the strongest binding affinities to IL18R1, with binding energies of −7.489 kcal/mol and −7.416 kcal/mol, respectively, both below the threshold of −7.0 kcal/mol, indicating robust interactions. Dehydrocostus lactone demonstrated notable binding activity to CCL11, with a binding energy of −6.320 kcal/mol. Vasicine hydrochloride showed significant binding capabilities to both CCL11 and MCP2, with binding energies of −5.830 kcal/mol and −5.008 kcal/mol, respectively, highlighting its potential for multi-target engagement. Raspberry ketone glucoside exhibited stable binding activity to both IL18R1 and MCP2. All molecular docking results are presented in Supplementary Figure S3. Through the integration of deep learning screening and molecular docking validation, we screened several small molecule candidates with high binding affinity for inflammatory targets, thereby providing a theoretical foundation for future experimental investigations. Notably, none of the shortlisted compounds are approved by the U.S. Food and Drug Administration (FDA); instead, they are primarily research/natural-product compounds, with antroquinonol being clinical-stage and having an FDA orphan drug designation.

**Table 3:** Deep learning and molecular docking results of selected small molecules with target proteins.

Proteins	Small molecules	Deep learning score	Binding energy (kcal/mol)
CCL11	Vasicine hydrochloride	0.993	−5.830
	Antroquinonol	0.991	−6.159
	Dehydrocostus lactone	0.985	−6.320
IL18R1	Dracorhodin perchlorate	0.998	−7.416
	Pseudolaric acid A-O-β-D-glucopyranoside	0.992	−7.489
	Raspberry ketone glucoside	0.992	−6.008
MCP2	Vasicine hydrochloride	0.991	−5.008
	Dehydrocostuslactone	0.978	−5.819
	Raspberry ketone glucoside	0.977	−5.693

Deep learning scores range from 0 to 1, with higher scores indicating a greater probability of binding.

## 4 Discussion

Using large-scale publicly available GWAS data, we applied Mendelian randomization to examine bidirectional, suggestive causal relationships between 91 circulating inflammatory proteins and SFN. We observed suggestive associations indicating that genetically predicted levels of CCL11 and IL18R1 were linked to increased SFN risk, whereas MCP2 showed a suggestive protective association. After BH-FDR correction across 91 proteins, none of the associations met the adjusted significance threshold; therefore, these signals were interpreted as suggestive candidates and were prioritized for downstream validation. SFN was associated with reduced CD6 levels, with no evidence supporting bidirectional causality. Colocalization analysis suggested a moderate association between the genetic instrument for CCL11 and SFN. Accordingly, we tone down causal interpretation and treat these results as suggestive genetic signals, as colocalization support was moderate for CCL11 (PPH4 = 0.612) and weak for IL18R1 (PPH4 = 0.100) and MCP2 (PPH4 = 0.251), which limits evidence for a shared causal variant. Through deep learning–based screening and molecular docking, we screened multiple suggestive candidate compounds for further evaluation.

As understanding of SFN has advanced, it has become increasingly recognized in clinical practice. Peripheral neuropathy affects an estimated 15–20 million Americans over the age of 40 and is associated with substantial healthcare costs (Tavee and Zhou, 2009). SFN can result from metabolic, toxic, immune-mediated, and genetic factors (Sène, 2018). Skin biopsy for IENFD assessment, Quantitative Sudomotor Axon Reflex Testing, and additional ancillary techniques are utilized in diagnosing SFN (Raasing et al., 2021). Nevertheless, the etiologic complexity of SFN and limitations of current diagnostic pathways make accurate diagnosis and differential diagnosis challenging, and a considerable number of patients may remain undiagnosed.

The pathophysiology and mechanisms of small fiber neuropathy are not yet fully understood. Emerging evidence suggests a close relationship between inflammatory proteins and SFN (Kreß et al., 2021; Kreß et al., 2023). Patients with SFN often suffer from burning pain in the extremities, and circulating inflammatory proteins are associated with diabetic peripheral neuropathy, visceral pain, injury to the peripheral nervous system and damage to the central nervous system (Lu et al., 2017; Ciechanowska and Mika, 2024; Zhou and Zhou, 2014). However, it remains unclear which inflammatory proteins may contribute to SFN risk, which may be protective, and which changes might occur secondary to SFN. Clarifying the causal relationships between circulating inflammatory proteins and SFN is therefore essential for a more comprehensive understanding of SFN pathogenesis.

CCL11, a member of the CC chemokine family, primarily signals through C-C chemokine receptor 3 (CCR3) and C-C chemokine receptor 5 (CCR5), regulating the activation and migration of eosinophils, basophils, neutrophils, and macrophages. CCL11 has been implicated in eosinophil-related disorders such as asthma, allergic rhinitis, and other allergic conditions (Xu and Su, 2023; Gazzinelli-Guimaraes et al., 2023). Research indicates that by enhancing reactive oxygen species production in microglia, CCL11 could induce neuronal cytotoxicity (Parajuli et al., 2015); In a rat model with chronic sciatic nerve constriction injury, elevated CCL11 protein levels were observed in the DRG, and blocking CCR3 was found to increase the analgesic effects of morphine and buprenorphine (Pawlik et al., 2021). Research indicates that CCL11 may play a role in nerve injury, and blocking its interaction with receptors might decrease the activation and migration of related inflammatory cells. CCL11 demonstrates suggestive interactions with therapeutic targets associated with SFN. These interactions primarily involve specific signaling pathways that may be relevant to pathogenesis of SFN, highlighting CCL11’s potential role in disease mechanisms and its relevance as a therapeutic point of interest. In our study, CCL11 showed suggestive links to SFN-related targets and pathways that may be relevant to SFN pathogenesis, highlighting CCL11 as a candidate of interest for future mechanistic and therapeutic investigation.

IL18R1 is a receptor for the pro-inflammatory cytokine IL-18 and contributes to inflammatory responses, including the recruitment and activation of neutrophils and the subsequent release of inflammatory mediators (Ihim et al., 2022). Genetic evidence has suggested a suggestive causal relationship between higher genetically predicted IL-18 levels and increased risk of inflammatory bowel disease and related subtypes, implying that disrupting IL-18–IL18R1 signaling may reduce susceptibility to inflammatory conditions (Mi et al., 2022). IL18R1 may represent a suggestive therapeutic target for SFN that warrants further investigation.

MCP2, also known as C-C motif chemokine ligand 8 (CCL8), is a member of the CC chemokine family and plays an important role in monocyte recruitment and inflammatory responses. CCL8 levels in cerebrospinal fluid are significantly elevated in patients with neuropathic pain or cluster headache (Bäckryd et al., 2017; Ran et al., 2024). However, the role of CCL8 in brain injury and peripheral neuropathic pain is generally unknown. Animal studies suggest that CCL8 contributes to increased sensitivity to thermal and mechanical stimuli and might contribute to neuropathic pain after chronic constriction injury of the sciatic nerve in mice (Pawlik et al., 2022); In addition, CCL8/CCR5 signaling in the spinal cord has been implicated in visceral pain hypersensitivity in murine colitis models (Lu et al., 2017). Because the homology between human and murine CCL8 is relatively low (Nomiyama et al., 2010; Naderi et al., 2022), caution is advised when extrapolating findings from murine models to human conditions and evaluating its significance in human pathologies. MR analysis suggested a potential protective association of CCL8 on SFN. Future studies are needed to investigate this potential causal relationship and clarify the underlying mechanisms.

The paclitaxel-induced mouse SFN model provides supportive experimental context for the MR-based prioritization. In these mice, CCL11, IL18R1, and MCP2 were upregulated in serum, dorsal root ganglia, and spinal cord. The changes in CCL11 and IL18R1 are directionally consistent with their MR-based risk associations, whereas MCP2 shows an apparent discordance with its suggestive protective association in MR. MCP2 represents an interesting case where genetic and experimental signals may not align in direction. The MR estimate reflects the average effect of genetically predicted, long-term differences in circulating MCP2 on SFN risk, whereas the PTX model captures state-dependent responses to an acute neurotoxic insult. Thus, MCP2 upregulation in mice may reflect a reactive or compensatory program engaged during nerve injury and inflammation rather than a causal risk mechanism. In addition, inflammatory signaling is highly context dependent: MCP2 regulation may vary across tissues (e.g., circulation, DRG, spinal cord, skin), cellular sources, and stages of CIPN/SFN progression. Collectively, these considerations constrain causal interpretation and suggest that MCP2 should be viewed as a prioritized, suggestive candidate that warrants further mechanistic dissection in future work (e.g., temporal profiling and cell-type–resolved analyses).

From a translational perspective, our deep learning–assisted screening nominated candidate compounds with high predicted interaction likelihood for prioritized targets. Molecular docking yielded binding energies below −7.0 kcal/mol for selected candidates, providing suggestive, hypothesis-generating support for plausible target–compound interactions and a starting point for future validation. This integrated computational and experimental framework provides insights into SFN-related inflammatory mechanisms and highlights candidates for follow-up therapeutic investigation.

### 4.1 Limitations of this study

A main strength of our study is the use of Mendelian randomization to systematically examine bidirectional, suggestive causal relationships between SFN and 91 circulating inflammatory proteins. Our study has several limitations: (1) We adopted a relatively lenient instrument-selection threshold (*p* < 5 × 10^−6^) and used *p* < 1 × 10^−5^ for reverse MR to ensure an adequate number of instruments; while this improves instrument availability, it may increase the risk of false-positive findings and should be interpreted cautiously. (2) As with all Mendelian randomization analyses, the core assumptions—relevance, independence, and exclusion restriction—cannot be fully verified. Although we performed a series of sensitivity analyses, including heterogeneity testing (Cochran’s Q), pleiotropy assessment (MR-Egger), outlier detection/correction (MR-PRESSO), and leave-one-out analyses, residual pleiotropy or other violations may still introduce bias. (3) The GWAS data were predominantly derived from individuals of European ancestry, which may limit generalizability to other populations. (4) While we observed expression changes of prioritized inflammatory factors in a paclitaxel-induced mouse model, these state-dependent alterations provide supportive context rather than definitive evidence of causality, and further mechanistic validation using multidimensional approaches is warranted. (5) our docking results (e.g., binding energies < −7 kcal/mol) are suggestive and were used to screen candidates; however, we did not perform molecular dynamics simulations or binding-site validation, and therefore these predictions should not be interpreted as confirmed binding or therapeutic efficacy. (6) We did not directly quantify the prioritized inflammatory protein targets in skin lysates. Given the distal predominance of painful CIPN/SFN and the importance of the cutaneous terminal-nerve microenvironment, this is an important limitation. (7) Our protein measurements were obtained within a single post-treatment time window and we did not evaluate these targets at multiple time points representing distinct phases of CIPN progression (e.g., initiation, maintenance, and resolution). Because molecular signatures can vary across stages, future studies incorporating longitudinal sampling—particularly in distal skin and terminal nerve compartments—will be important to define the temporal and tissue-specific roles of these targets.

## 5 Conclusion

This study employed a comprehensive approach, integrating Mendelian randomization analysis, animal experimentation, and computational biology, to provide support for specific inflammatory factors in SFN. Genetic analyses nominated CCL11 and IL18R1 suggestive as risk factors for SFN, whereas MCP2 demonstrated a suggestive protective effect at the genetic level. These genetic findings were investigated by a paclitaxel-induced neuropathy model, which provided support for the upregulation of CCL11 and IL18R1 during disease progression. However, MCP2 showed discordant genetic and experimental signals, which may reflect context-dependent regulation and differences between genetically predicted long-term effects and acute injury responses. Furthermore, deep learning–assisted screening nominated candidate compounds with high predicted interaction likelihood for targets such as CCL11 and IL18R1. Together with docking results, these findings provide suggestive leads for future validation. This integrative investigation advances the understanding of SFN pathogenesis and highlights directions for subsequent therapeutic research.

## Data Availability

The datasets presented in this study can be found in online repositories. The names of the repository/repositories and accession number(s) can be found in the article/Supplementary material.

## Footnotes

1
https://www.finngen.fi/en/access_results

2
https://ldlink.nih.gov/?tab=ldtrait

3
https://azphewas.com/

4
https://www.targetmol.com

5
https://www.uniprot.org/

6
https://pubchem.ncbi.nlm.nih.gov/

7
https://www.rcsb.org/

8
https://www.r-project.org

## References

[ref1] Bäckryd E. Lind A. L. Thulin M. Larsson A. Gerdle B. Gordh T. (2017). High levels of cerebrospinal fluid chemokines point to the presence of neuroinflammation in peripheral neuropathic pain: a cross-sectional study of 2 cohorts of patients compared with healthy controls. Pain 158, 2487–2495. doi: 10.1097/j.pain.0000000000001061, 28930774 PMC5690569

[ref2] Bautista J. Chandrasekhar A. Komirishetty P. K. Duraikannu A. Zochodne D. W. (2020). Regenerative plasticity of intact human skin axons. J. Neurol. Sci. 417:117058. doi: 10.1016/j.jns.2020.117058 32755738

[ref3] Burgess S. Davey Smith G. Davies N. M. Dudbridge F. Gill D. Glymour M. M. . (2019). Guidelines for performing Mendelian randomization investigations: update for summer 2023. Wellcome Open Res. 4:186. doi: 10.12688/wellcomeopenres.15555.3, 32760811 PMC7384151

[ref4] Burgess S. Thompson S. G. (2017). Interpreting findings from Mendelian randomization using the MR-egger method. Eur. J. Epidemiol. 32, 377–389. doi: 10.1007/s10654-017-0255-x, 28527048 PMC5506233

[ref5] Chen L. Fan Z. Chang J. Yang R. Hou H. Guo H. . (2023). Sequence-based drug design as a concept in computational drug design. Nat. Commun. 14:4217. doi: 10.1038/s41467-023-39856-w, 37452028 PMC10349078

[ref6] Ciechanowska A. Mika J. (2024). CC chemokine family members' modulation as a novel approach for treating central nervous system and peripheral nervous system injury-a review of clinical and experimental findings. Int. J. Mol. Sci. 25:3788. doi: 10.3390/ijms25073788, 38612597 PMC11011591

[ref7] Devigili G. Cazzato D. Lauria G. (2020). Clinical diagnosis and management of small fiber neuropathy: an update on best practice. Expert. Rev. Neurother. 20, 967–980. doi: 10.1080/14737175.2020.1794825, 32654574

[ref8] Doncheva N. T. Morris J. H. Holze H. Kirsch R. Nastou K. C. Cuesta-Astroz Y. . (2023). Cytoscape stringApp 2.0: analysis and visualization of heterogeneous biological networks. J. Proteome Res. 22, 637–646. doi: 10.1021/acs.jproteome.2c00651, 36512705 PMC9904289

[ref9] Emdin C. A. Khera A. V. Kathiresan S. (2017). Mendelian randomization. JAMA 318, 1925–1926. doi: 10.1001/jama.2017.17219, 29164242

[ref10] Gazzinelli-Guimaraes P. H. Golec D. P. Karmele E. P. Sciurba J. Bara-Garcia P. Hill T. . (2023). Eosinophil trafficking in allergen-mediated pulmonary inflammation relies on IL-13-driven CCL-11 and CCL-24 production by tissue fibroblasts and myeloid cells. J. Allergy Clin. Immunol. Glob. 2:100131. doi: 10.1016/j.jacig.2023.100131, 37781651 PMC10509988

[ref11] Gross F. Üçeyler N. (2020). Mechanisms of small nerve fiber pathology. Neurosci. Lett. 737:135316. doi: 10.1016/j.neulet.2020.135316 32828814

[ref12] Gupta P. Makkar T. K. Goel L. Pahuja M. (2022). Role of inflammation and oxidative stress in chemotherapy-induced neurotoxicity. Immunol. Res. 70, 725–741. doi: 10.1007/s12026-022-09307-7, 35859244

[ref13] Ihim S. A. Abubakar S. D. Zian Z. Sasaki T. Saffarioun M. Maleknia S. . (2022). Interleukin-18 cytokine in immunity, inflammation, and autoimmunity: biological role in induction, regulation, and treatment. Front. Immunol. 13:919973. doi: 10.3389/fimmu.2022.919973, 36032110 PMC9410767

[ref14] Jia R. Wan L. Jin L. Tian Q. Chen Y. Zhu X. . (2025). Fucoidan reduces NET accumulation and alleviates chemotherapy-induced peripheral neuropathy via the gut-blood-DRG axis. J. Neuroinflammation 22:100. doi: 10.1186/s12974-025-03431-5, 40186245 PMC11969723

[ref15] Kelley M. A. Hackshaw K. V. (2021). Intraepidermal nerve fiber density as measured by skin punch biopsy as a marker for small Fiber neuropathy: application in patients with fibromyalgia. Diagnostics (Basel) 11:536. doi: 10.3390/diagnostics11030536, 33802768 PMC8002511

[ref16] Kreß L. Egenolf N. Sommer C. Üçeyler N. (2023). Cytokine expression profiles in white blood cells of patients with small fiber neuropathy. BMC Neurosci. 24:1. doi: 10.1186/s12868-022-00770-4, 36604634 PMC9817338

[ref17] Kreß L. Hofmann L. Klein T. Klug K. Saffer N. Spitzel M. . (2021). Differential impact of keratinocytes and fibroblasts on nociceptor degeneration and sensitization in small fiber neuropathy. Pain 162, 1262–1272. doi: 10.1097/j.pain.0000000000002122, 33196576

[ref18] Kurki M. I. Karjalainen J. Palta P. Sipilä T. P. Kristiansson K. Donner K. M. . (2023). FinnGen provides genetic insights from a well-phenotyped isolated population. Nature 613, 508–518. doi: 10.1038/s41586-022-05473-8, 36653562 PMC9849126

[ref19] Lu Y. Jiang B. C. Cao D. L. Zhao L. X. Zhang Y. L. (2017). Chemokine CCL8 and its receptor CCR5 in the spinal cord are involved in visceral pain induced by experimental colitis in mice. Brain Res. Bull. 135, 170–178. doi: 10.1016/j.brainresbull.2017.10.009 29037608

[ref20] Mi J. Liu Z. Pei S. Wu X. Zhao N. Jiang L. . (2022). Mendelian randomization study for the roles of IL-18 and IL-1 receptor antagonist in the development of inflammatory bowel disease. Int. Immunopharmacol. 110:109020. doi: 10.1016/j.intimp.2022.109020, 35843146

[ref21] Naderi A. Farmaki E. Chavez B. Cai C. Kaza V. Zhang Y. . (2022). Beneficial effects of CCL8 inhibition at lipopolysaccharide-induced lung injury. iScience 25:105520. doi: 10.1016/j.isci.2022.105520, 36404927 PMC9639378

[ref22] Nomiyama H. Osada N. Yoshie O. (2010). The evolution of mammalian chemokine genes. Cytokine Growth Factor Rev. 21, 253–262. doi: 10.1016/j.cytogfr.2010.03.004, 20434943

[ref23] Oaklander A. L. Nolano M. (2019). Scientific advances in and clinical approaches to small-Fiber polyneuropathy: a review. JAMA Neurol. 76, 1240–1251. doi: 10.1001/jamaneurol.2019.2917, 31498378 PMC10021074

[ref24] Parajuli B. Horiuchi H. Mizuno T. Takeuchi H. Suzumura A. (2015). CCL11 enhances excitotoxic neuronal death by producing reactive oxygen species in microglia. Glia 63, 2274–2284. doi: 10.1002/glia.22892, 26184677

[ref25] Pawlik K. Ciapała K. Ciechanowska A. Kwiatkowski K. Mika J. (2022). Pharmacological evidence of the important roles of CCR1 and CCR3 and their endogenous ligands CCL2/7/8 in hypersensitivity based on a murine model of neuropathic pain. Cells 12:98. doi: 10.3390/cells12010098, 36611891 PMC9818689

[ref26] Pawlik K. Ciechanowska A. Ciapała K. Rojewska E. Makuch W. Mika J. (2021). Blockade of CC chemokine receptor type 3 diminishes pain and enhances opioid analgesic potency in a model of neuropathic pain. Front. Immunol. 12:781310. doi: 10.3389/fimmu.2021.781310, 34795678 PMC8593225

[ref27] Raasing L. R. M. Vogels O. J. M. Veltkamp M. Van Swol C. F. P. Grutters J. C. (2021). Current view of diagnosing small Fiber neuropathy. J. Neuromuscul. Dis. 8, 185–207. doi: 10.3233/jnd-200490, 33337383 PMC8075405

[ref28] Ran C. Olofsgård F. J. Wellfelt K. Steinberg A. Belin A. C. (2024). Elevated cytokine levels in the central nervous system of cluster headache patients in bout and in remission. J. Headache Pain 25:121. doi: 10.1186/s10194-024-01829-9, 39044165 PMC11267889

[ref29] Sankaranarayanan I. Tavares-Ferreira D. Mwirigi J. M. Mejia G. L. Burton M. D. Price T. J. (2023). Inducible co-stimulatory molecule (ICOS) alleviates paclitaxel-induced neuropathic pain via an IL-10-mediated mechanism in female mice. J. Neuroinflammation 20:32. doi: 10.1186/s12974-023-02719-8, 36774519 PMC9922469

[ref30] Sène D. (2018). Small fiber neuropathy: diagnosis, causes, and treatment. Joint Bone Spine 85, 553–559. doi: 10.1016/j.jbspin.2017.11.002, 29154979

[ref31] Sharma S. Vas P. Rayman G. (2022). Small Fiber neuropathy in diabetes polyneuropathy: is it time to change? J. Diabetes Sci. Technol. 16, 321–331. doi: 10.1177/1932296821996434, 33840265 PMC8861803

[ref32] Szklarczyk D. Kirsch R. Koutrouli M. Nastou K. Mehryary F. Hachilif R. . (2023). The STRING database in 2023: protein-protein association networks and functional enrichment analyses for any sequenced genome of interest. Nucleic Acids Res. 51, D638–d646. doi: 10.1093/nar/gkac1000, 36370105 PMC9825434

[ref33] Tavee J. (2022). Peripheral neuropathy in sarcoidosis. J. Neuroimmunol. 368:577864. doi: 10.1016/j.jneuroim.2022.577864, 35585009

[ref34] Tavee J. Zhou L. (2009). Small fiber neuropathy: a burning problem. Cleve. Clin. J. Med. 76, 297–305. doi: 10.3949/ccjm.76a.08070, 19414545

[ref35] Terkelsen A. J. Karlsson P. Lauria G. Freeman R. Finnerup N. B. Jensen T. S. (2017). The diagnostic challenge of small fibre neuropathy: clinical presentations, evaluations, and causes. Lancet Neurol. 16, 934–944. doi: 10.1016/s1474-4422(17)30329-0, 29029847

[ref36] Timmins H. C. Li T. Kiernan M. C. Horvath L. G. Goldstein D. Park S. B. (2020). Quantification of small Fiber neuropathy in chemotherapy-treated patients. J. Pain 21, 44–58. doi: 10.1016/j.jpain.2019.06.011, 31325646

[ref37] Trott O. Olson A. J. (2010). AutoDock vina: improving the speed and accuracy of docking with a new scoring function, efficient optimization, and multithreading. J. Comput. Chem. 31, 455–461. doi: 10.1002/jcc.21334, 19499576 PMC3041641

[ref38] Wang Q. Dhindsa R. S. Carss K. Harper A. R. Nag A. Tachmazidou I. . (2021). Rare variant contribution to human disease in 281,104 UK biobank exomes. Nature 597, 527–532. doi: 10.1038/s41586-021-03855-y, 34375979 PMC8458098

[ref39] Woolf C. J. Salter M. W. (2000). Neuronal plasticity: increasing the gain in pain. Science 288, 1765–1768. doi: 10.1126/science.288.5472.1765, 10846153

[ref40] Xu C. Su W. (2023). Hyperforin modulates MAPK/CCL11 signaling to reduce the inflammatory response of nasal mucosal epithelial cells caused by allergic rhinitis by targeting BCL6. Exp. Ther. Med. 26:579. doi: 10.3892/etm.2023.12278, 38023351 PMC10655049

[ref41] Zhao J. H. Stacey D. Eriksson N. Macdonald-Dunlop E. Hedman Å K. Kalnapenkis A. . (2023). Genetics of circulating inflammatory proteins identifies drivers of immune-mediated disease risk and therapeutic targets. Nat. Immunol. 24, 1540–1551. doi: 10.1038/s41590-023-01588-w 37563310 PMC10457199

[ref42] Zhou J. Zhou S. (2014). Inflammation: therapeutic targets for diabetic neuropathy. Mol. Neurobiol. 49, 536–546. doi: 10.1007/s12035-013-8537-0, 23990376

