# Supplementary material for: Identifying potential inflammatory therapeutic targets and drug candidates in small fiber neuropathy: integrating Mendelian randomization, experimental validation, and deep learning
Source: Front Neurosci. 2026 Mar 24;20:1781396. doi: 10.3389/fnins.2026.1781396 (PMC13055505; doi:10.3389/fnins.2026.1781396)
Supplement: Supplementary file 1 [file Data_sheet_1.docx]

Supplementary Material

1. **Supplementary Figures**

## **1.1 Supplementary Figures**

**
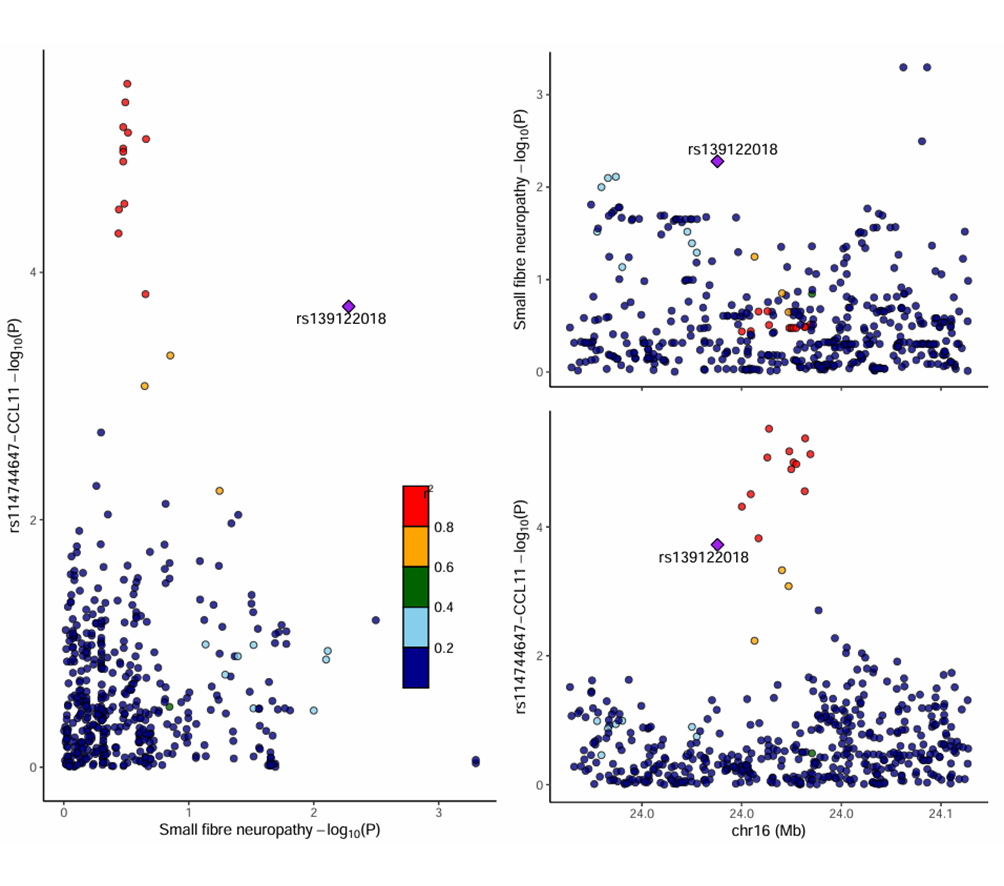
**

**Supplementary Figure 1.** Analysis of the co-localization of CCL11 with small fiber neuropathy.


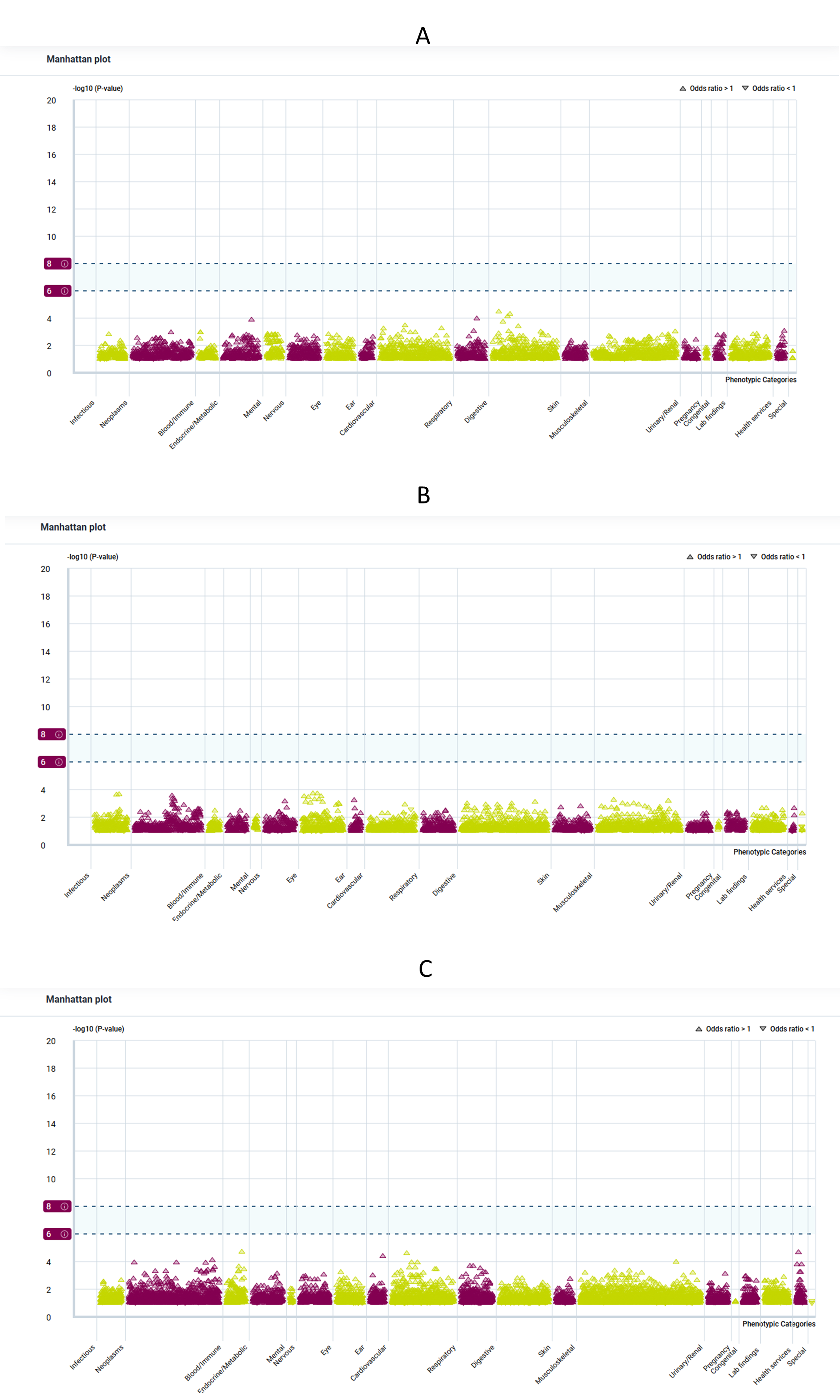


**Supplementary Figure 2.** The result of a phenome-wide association study for CCL11 (A), MCP2 (B) and IL18R1 (C).


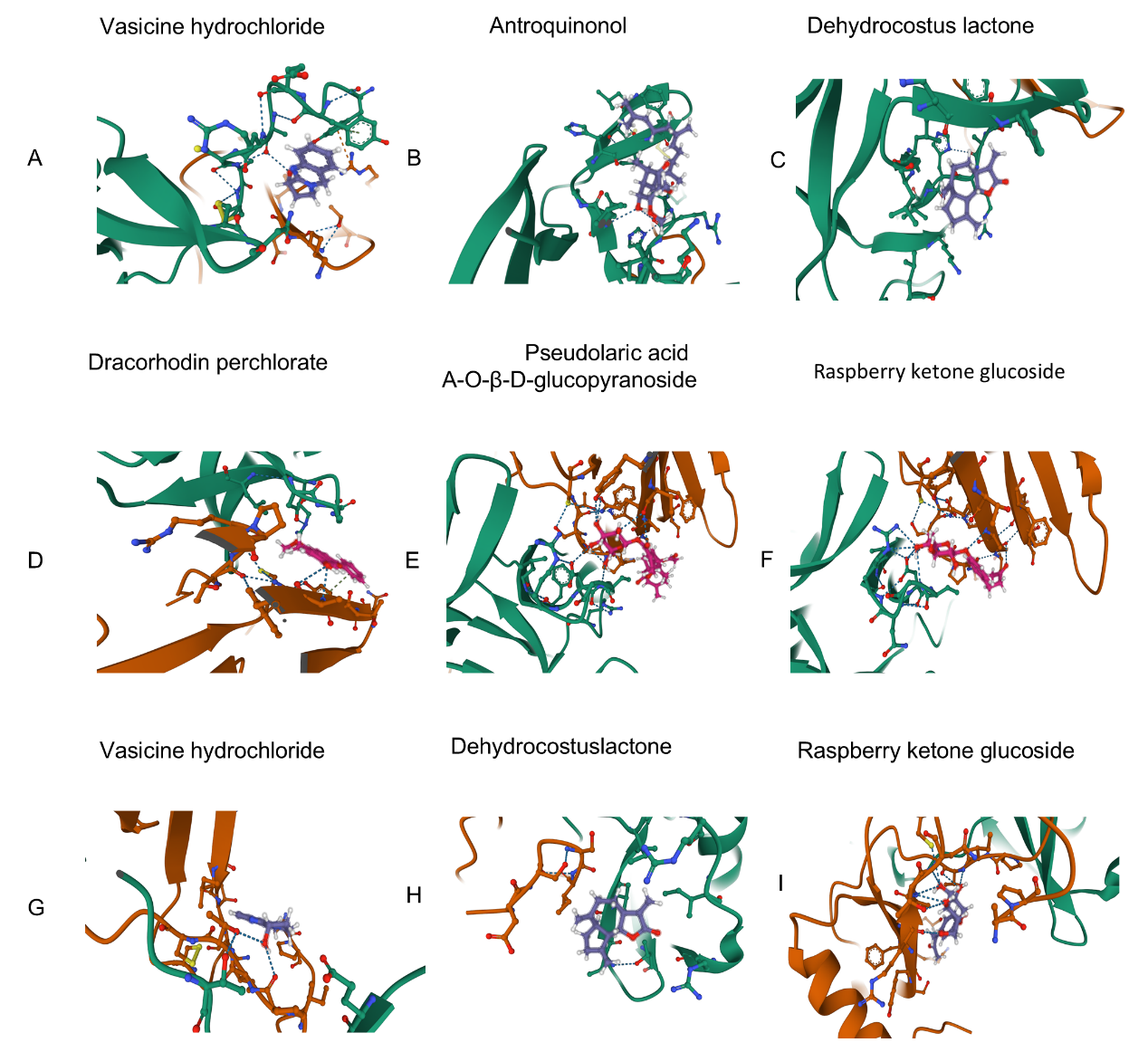


**Supplementary Figure 3.** The outcome of molecular docking involving drugs and three inflammatory proteins: (A) Vasicine hydrochloride docking with CCL11; (B) Antroquinonol docking with CCL11; (C)Dehydrocostus lactone docking with CCL11; (D) Dracorhodin perchlorate docking with IL18R1; (E)Pseudolaric acid A-O-β-D-glucopyranoside docking with IL18R1; (F) Raspberry ketone glucoside docking with IL18R1. (G) Vasicine hydrochloride docking with MCP2. (H) Dehydrocostuslactone docking with MCP2. (I) Raspberry ketone glucoside docking with MCP2.
